# Reducing infections from central lines in a neonatal intensive care unit, Egypt

**DOI:** 10.2471/BLT.24.291949

**Published:** 2025-04-01

**Authors:** Hoda Mohamed Owais, Nesrine Fathi Hanafi, Ghada Abdel- Wahed Ismail, Moustapha Ramadan

**Affiliations:** a Faculty of Medicine, Alexandria University, Champollion Street, Azarita, 21526 Alexandria, Egypt.; b Faculty of Medicine, Ain Shams University, Cairo, Egypt.

## Abstract

**Problem:**

Central line-associated bloodstream infections in critically ill neonates are major challenge in neonatal intensive care units.

**Approach:**

In April 2023, a multidisciplinary team, consisting of the infection prevention and control team, the unit head, a neonatal consultant doctor, a senior doctor and a head nurse, introduced the World Health Organization Multimodal Hand Hygiene Improvement Strategy in the neonatal intensive care unit of El-Shatby University Hospital, Egypt. The team introduced an antiseptic handwash and a disinfectant for surfaces and equipment, especially incubators. To highlight the incidence of infections in the unit and illustrate the effectiveness of the newly introduced products, the team offered training programmes for all health workers. Health workers’ proper use of the introduced products was monitored and, if necessary, immediate corrective actions were taken. Monthly meetings were held to discuss hand hygiene compliance, infection rates and challenges in infection prevention and control.

**Local setting:**

The neonatal intensive care unit has 70 incubators and 28 beds.

**Relevant changes:**

The central line-associated bloodstream infection rate decreased from 13.85 infections per 1000 device days (95% confidence interval, CI: 10.44–18.03) before the intervention to 9.08 infections per 1000 device days (95% CI: 5.81–11.27). Hand hygiene compliance increased from 58% (70/120) to 71% (88/124) among nurses and from 64% (58/91) to 67% (67/100) among doctors.

**Lessons learnt:**

Implementing a multimodal strategy through a multidisciplinary approach led to positive changes in infection prevention and control practices, and a reduction in central line bloodstream infections.

## Résumé

**Problème:**

Les infections sanguines associées aux cathéters centraux chez les nouveau-nés gravement malades constituent un défi majeur dans les unités de soins intensifs néonatals.

**Approche:**

En avril 2023, une équipe multidisciplinaire composée de l’équipe de prévention et de contrôle des infections, du chef d’unité, d’un médecin consultant en néonatalogie, d’un médecin en chef et d’une infirmière en chef, a lancé la stratégie multimodale de l’Organisation mondiale de la santé pour la promotion de l’hygiène des mains au sein de l’unité de soins intensifs néonatals du centre hospitalier universitaire El-Shatby, en Égypte. L’équipe a introduit un lavage antiseptique des mains et un désinfectant pour les surfaces et les équipements, en particulier les couveuses. Pour mettre en évidence l’incidence des infections au sein de leur unité et pour illustrer l’efficacité des produits nouvellement introduits, l’équipe a proposé des programmes de formation à l’ensemble du personnel de santé. L’utilisation correcte des produits introduits par les travailleurs de la santé a été contrôlée et, le cas échéant, des mesures correctives immédiates ont été prises. Des réunions mensuelles ont été organisées pour discuter du respect de l’hygiène des mains, des taux d’infection et des défis à relever en matière de prévention et de contrôle des infections.

**Environnement local:**

L’unité de soins intensifs néonatals dispose de 70 couveuses et de 28 lits.

**Changements significatifs:**

Le taux d’infections sanguines liées aux cathéters centraux est passé de 13,85 infections pour 1 000 jours de fonctionnement (intervalle de confiance (IC) à 95%: 10,44–18,03) avant l’intervention à 9,08 infections pour 1 000 jours de fonctionnement (IC à 95%: 5,81–11,27). L’observance de l’hygiène des mains est passée de 58% (70/120) à 71% (88/124) chez le personnel infirmier et de 64% (58/91) à 67% (67/100) chez les médecins.

**Leçons tirées:**

La mise en œuvre d’une stratégie multimodale dans le cadre d’une approche multidisciplinaire a entraîné des changements positifs dans les pratiques de prévention et de contrôle des infections, ainsi qu’une réduction des infections sanguines liées aux cathéters centraux.

## Resumen

**Situación:**

Las infecciones del torrente sanguíneo asociadas a catéteres centrales en recién nacidos críticamente enfermos constituyen un importante reto en las unidades de cuidados intensivos neonatales.

**Enfoque:**

En abril de 2023, un equipo multidisciplinar, compuesto por el equipo de prevención y control de infecciones, el jefe de unidad, un médico consultor en neonatología, un médico sénior y el enfermero jefe, implementó la Estrategia multimodal de mejora de la higiene de manos de la Organización Mundial de la Salud en la unidad de cuidados intensivos neonatales del Hospital Universitario El-Shatby, en Egipto. Se introdujo un producto antiséptico para el lavado de manos y un desinfectante para superficies y equipos, en especial incubadoras. Con el objetivo de visibilizar la incidencia de infecciones en la unidad y demostrar la eficacia de los productos incorporados, se ofrecieron programas de formación para todo el personal sanitario. Se supervisó el uso adecuado de los productos por parte del personal y, en caso necesario, se adoptaron medidas correctivas inmediatas. Se celebraron reuniones mensuales para analizar el cumplimiento de la higiene de manos, las tasas de infección y los desafíos en prevención y control de infecciones.

**Marco regional:**

La unidad de cuidados intensivos neonatales cuenta con 70 incubadoras y 28 camas.

**Cambios importantes:**

La tasa de infecciones del torrente sanguíneo asociadas a catéteres centrales se redujo de 13,85 infecciones por cada 1000 días de uso del dispositivo (intervalo de confianza [IC] del 95%: 10,44–18,03) antes de la intervención a 9,08 infecciones por cada 1000 días de uso del dispositivo (IC del 95%: 5,81–11,27). El cumplimiento de la higiene de manos aumentó del 58% (70/120) al 71% (88/124) entre el personal de enfermería, y del 64% (58/91) al 67% (67/100) entre el personal médico.

**Lecciones aprendidas:**

La implementación de una estrategia multimodal mediante un enfoque multidisciplinar condujo a mejoras en las prácticas de prevención y control de infecciones, así como a una reducción de las infecciones del torrente sanguíneo asociadas a catéteres centrales.

## ملخص

المشكلة إن حالات العدوى في مجرى الدم المرتبطة بالخطوط المركزية لدى حديثي الولادة ذوي الحالات الحرجة، تمثل تحديًا رئيسيًا في وحدات العناية المركزة لحديثي الولادة.

الأسلوب في أبريل/نيسان 2023، قام فريق متعدد التخصصات، يتألف من فريق الوقاية من العدوى ومكافحتها، ورئيس الوحدة، وطبيب استشاري لحديثي الولادة، وطبيب أول، ورئيسة ممرضات، بتطبيق "استراتيجية منظمة الصحة العالمية لتحسين نظافة اليدين متعددة الوسائط" في وحدة العناية المركزة لحديثي الولادة بمستشفى الشاطبي الجامعي في مصر. قام الفريق بطرح غسول مطهر لليدين ومطهر للأسطح والمعدات، وخاصةً الحضّانات. ولتسليط الضوء على حدوث العدوى في الوحدة، وتوضيح فعالية المنتجات المطروحة مؤخرًا، قام الفريق بتقديم برامج تدريبية لجميع العاملين في المجال الصحي. وتمت مراقبة الاستخدام السليم من جانب العاملين في المجال الصحي للمنتجات الجديدة، واتخاذ إجراءات تصحيحية فورية عند الضرورة. تم عقد اجتماعات شهرية لمناقشة الالتزام بنظافة اليدين، ومعدلات الإصابة، والتحديات في مجال الوقاية من العدوى ومكافحتها.

المواقع المحلية تحتوي وحدة العناية المركزة لحديثي الولادة على 70 حضّانة و28 سريرًا.

التغيّرات ذات الصلة انخفض معدل عدوى مجرى الدم المرتبطة بالخط المركزي من 13.85 عدوى لكل 1000 يوم استخدام للجهاز (بنسبة أرجحية مقدارها %95: 10.44 إلى 18.03) قبل التدخل إلى 9.08 عدوى لكل 1000 يوم استخدام للجهاز (بنسبة أرجحية مقدارها %95: 5.81 إلى 11.27). وارتفع مستوى الالتزام بنظافة اليدين من %58 (70/120) إلى %71 (88/124) بين الممرضات، ومن %64 (58/91) إلى %67 (67/100) بين الأطباء.

الدروس المستفادة إن تطبيق استراتيجية متعددة الوسائط من خلال أسلوب متعدد التخصصات، أدى إلى تغييرات إيجابية في ممارسات الوقاية من العدوى ومكافحتها، وانخفاض في عدوى مجرى الدم المرتبطة بالخط المركزي.

## 摘要

**问题:**

危重新生儿中心静脉导管相关血流感染是新生儿重症监护病房需面临的重大挑战。

**方法:**

2023 年 4 月，一个多学科小组（由感染预防和控制小组、组长、一名新生儿顾问医生、一名高级医生和一名护士长组成）将世界卫生组织多模式手卫生改善战略 (Multimodal Hand Hygiene Improvement Strategy) 引入了埃及埃尔沙特比大学附属医院 (El-Shatby University Hospital) 的新生儿重症监护病房。该小组引入了一种杀菌洗手液和一种消毒剂来为表面和设备（特别是新生儿保温箱）进行消毒处理。为了重点了解该病房的感染率并证明新引入产品的有效性，该小组制定了针对所有卫生工作者的培训方案。监测卫生工作者是否正确使用引入的产品，并在必要时立即采取纠正措施。每月召开会议，以讨论手卫生注意事项遵循情况、感染率以及在感染预防和控制方面所面临的挑战。

**当地状况:**

该新生儿重症监护病房共有 70 个新生儿保温箱和 28 张床位。

**相关变化:**

中心静脉导管相关血流感染率从干预前的每 1,000 个设备使用日 13.85 例感染【95% 置信区间 (CI)：10.44-18.03】降至了每 1,000 个设备使用日 9.08 例感染（95% CI：5.81-11.27）。遵循手卫生注意事项的护士比例从 58% (70/120) 上升至了 71% (88/124)，而遵循此类注意事项的医生比例也从 64% (58/91) 上升至了 67% (67/100)。

**经验教训:**

通过采用多学科方法实施多模式战略，感染预防和控制实践发生了积极改变，而中心静脉导管相关血流感染也有所减少。

## Резюме

**Проблема:**

Серьезную проблему для отделений интенсивной терапии новорожденных представляют инфекции циркулирующей крови у тяжелобольных пациентов, связанные с использованием центрального катетера.

**Подход:**

В апреле 2023 года мультидисциплинарная группа, состоящая из группы по профилактике и контролю инфекций, заведующего отделением, консультанта-неонатолога, главного врача и старшей медсестры, внедрила мультимодальную стратегию улучшения гигиены рук, разработанную Всемирной организацией здравоохранения, в отделении интенсивной терапии новорожденных в больнице университета Эль-Шатби в Египте. Группа внедрила антисептик для мытья рук и дезинфектант для обработки поверхностей и оборудования, в особенности инкубаторов. Чтобы привлечь внимание к частоте инфекций в отделении и проиллюстрировать эффективность вновь внедренных продуктов, группа предложила всем медработникам пройти учебные программы. В больнице следили за правильностью использования медработниками внедренной продукции и в случае необходимости немедленно принимали меры к исправлению замеченных недостатков. Ежемесячно организовывались рабочие встречи для обсуждения вопросов соблюдения гигиены, частоты возникновения инфекций, а также сложностей их профилактики и контроля.

**Местные условия:**

В отделении интенсивной терапии новорожденных имеется 70 инкубаторов и 28 коек.

**Осуществленные перемены:**

Частота инфекций циркулирующей крови, связанных с применением центрального катетера, снизилась с 13,85 случая на 1000 устройство-часов (95%-й доверительный интервал, ДИ: 10,44–18,03) перед вмешательством до 9,08 случая на 1000 устройство-часов (95%-й ДИ: 5,81–11,27). Соблюдение гигиены рук повысилось с 58% (70/120) до 71% (88/124) среди медсестер и с 64% (58/91) до 67% (67/100) среди врачей.

**Выводы:**

Внедрение мультимодальной стратегии на основе мультидисциплинарного подхода привело к положительным изменениям в части практик профилактики и контроля инфекций, а также снизило частоту инфекций циркулирующей крови, связанных с использованием центрального катетера.

## Introduction

Neonatal sepsis is one of the major challenges facing neonatal intensive care units in Egypt. Studies have shown that sepsis is diagnosed in 21.5% (153/711) to 45.9% (357/778) of admitted neonates in Egyptian university hospitals.^1^^,^^2^ In critically ill neonates, the insertion of a central venous catheter, called a central line, is a routine procedure that allows the administration of medications and parenteral nutrition. However, underlying illness, immature immune defence mechanisms and immature skin and intestinal barriers^2^ increase the risk of infection, making central line-associated bloodstream infections the most common health care-associated infections in neonatal intensive care units.^3^^–^^5^

Infection prevention and control strategies are highly effective in reducing health care-associated infections, as well as in decreasing morbidity and mortality in neonatal intensive care units.^6^ Among these strategies, hand hygiene is a key measure that improves patient safety, reducing infections, preventing antibiotic resistance and minimizing hospital stays and costs.^6^^,^^7^ Although hand hygiene is a simple practice, improving hand hygiene compliance requires comprehensive and integrated action plans.^6^ To support health-care facilities, the World Health Organization (WHO) has developed a multimodal hand hygiene improvement strategy.^8^^–^^10^ The strategy consists of five components: (i) system change; (ii) training and education; (iii) evaluation and feedback; (iv) reminders in the workplace; and (v) institutional safety climate.^8^

Here we describe how we implemented the WHO multimodal strategies to reduce the neonatal central line-associated bloodstream infections at a neonatal intensive care unit in Egypt. 

## Local setting

The neonatal intensive care unit at El Shatby University Hospital is one of the largest units in north-western Egypt, comprising 70 incubators, 28 beds, 25 doctors, 107 nurses and nine workers. Residents usually rotate every 6 months, which requires regular training and education. Doctors are responsible for inserting the central catheter, while nurses are responsible for the routine daily care of the catheter site thereafter.

The infection prevention and control team at the unit includes one qualified full-time doctor, two trained full-time nurses and one link nurse. The team is responsible for conducting regular morning rounds, infection surveillance, training staff, supervising clean and invasive procedures, and providing immediate corrective actions.

## Approach

To address the high number of laboratory-confirmed bloodstream infection, the infection prevention and control team at the neonatal intensive care unit started investigating the situation and planning for improvement interventions. During March 2023, several meetings were held with the head of the unit, resident doctors and head nurse to present data and emphasize the urgency of intervention to reduce infections. The team proposed and explained WHO’s multimodal approach, which subsequently the leadership chose as the interventional method.

In April 2023, a multidisciplinary team was created, consisting of the infection prevention and control team, the unit head, a neonatal consultant doctor, a senior doctor and a head nurse, and held several meetings to plan the implementation of the WHO multimodal strategy.

The implementation focused on three main actions for the system change. First, the infection prevention and control team obtained data from the Supreme Council of University Hospitals electronic surveillance system for device-associated infections. Using these data, the team sent official monthly reports of health care-associated infections and hand hygiene compliance to the unit head. Second, at the point of care, the multidisciplinary team replaced the regular handwash soap with a handwash product containing cetrimide 3.0% and chlorhexidine 0.3%, alongside the already available alcohol hand rub. This locally produced product is licensed by the Egyptian authorities for hand and skin cleansing purposes. Third, the multidisciplinary team took the opportunity during the implementation to also introduce a fourth generation of quaternary ammonium compound product for cleaning and disinfecting the unit’s surfaces and equipment, especially incubators. This licensed product, used in other university hospitals, meets the required European standards and is safe for use while the incubators are in operation, ensuring a clean environment for high-risk patients, especially those with invasive devices. The unit head sought approval for the use of both products from the general infection prevention and control supervisor and the director of the supply chain at the Alexandria University Hospitals, at no additional costs. The unit head instructed the staff to routinely use these available products as recommended by the manufacturers.

The infection prevention and control team conducted training programmes for all health workers, including structured lectures, to highlight the incidence of health care-associated infections in the unit and illustrate the effectiveness of the newly introduced products in infection prevention. All health workers received a practical demonstration and on-the-job training for correct and effective handwashing and hand rubbing. In addition, health workers responsible for disinfecting surfaces received training on the proper use, including dilution and storage, of the new disinfectant. Furthermore, special training for nurses focused on aseptic blood sampling and the correct method for blood culture.

The head nurse, as part of her daily routine, and a senior doctor during morning rounds monitored staff adherence to instructions and correct product use, and took immediate corrective actions if necessary. The infection prevention and control link nurse regularly monitored dispenser availability near neonatal incubators and regularly checked nurses' and doctors’ hand hygiene compliance, and provided unit heads with feedback. The infection prevention and control team met with the head nurse and senior doctors of the unit monthly to discuss hand hygiene compliance, rate of health care-associated infections, patterns of microorganisms detected in blood cultures, and infection prevention and control challenges. This information was also transmitted to the unit head through the official monthly reports.

To remind staff members to comply, posters for handwashing steps were present near the sinks. A chart was placed near the incubators to ensure they were properly disinfected. Illustrations of the correct dilution, concentration and storage period of different disinfectant products were clearly placed for end users.

We followed the case definition and surveillance forms of the Supreme Council of University Hospitals, which were adapted from the United States Centers for Disease Control and Prevention and the National Healthcare Safety Network case definitions for laboratory-confirmed bloodstream infections.^11^ We considered only laboratory-confirmed bloodstream infections occurring more than 72 hours after birth and that were associated with an indwelling central line in place for at least 48 hours. We excluded any early-onset bloodstream infection within 48 hours after admission to the neonatal intensive care unit, or only one positive blood culture considered to be skin colonizers, or any positive blood cultures proved to be secondary to another type of infection, as pneumonia. We obtained blood culture results and isolated microorganisms from microbiological surveillance laboratories. Alexandria University Hospitals funded the laboratory assessments. 

Other than staff time and fuel costs for picking up products at the main storage, no additional implementation costs were incurred. 

## Relevant changes

Between December 2022 and October 2023, 1162 neonates were admitted to the neonatal intensive care unit. While 581 neonates were admitted during both the pre- and post-intervention periods, the length of stay was higher in the post-intervention period (10 751 patient days versus 9583 in the pre-intervention period). Also, neonates had longer exposure to central line devices post-intervention (4625 central line days versus 3970 in the pre-intervention period). Of the 188 positive blood cultures recorded, 137 were primary bloodstream infections, of which 97 (55 pre-intervention and 42 post-intervention) were considered central line-associated bloodstream infections (Table 1). The most common group of microorganisms isolated in the blood cultures was Enterobacterales, especially *Klebsiella pneumoniae* (161 positive blood cultures). 

**Table 1:** Central line-associated bloodstream infections in a neonatal intensive care unit, before and after implementation of the WHO Multimodal Hand Hygiene Improvement Strategy, Egypt, December 2022 – October 2023

Variable	No. (%)^a^		*P*
	Pre-intervention	Post-intervention	
Positive blood culture	107 (56.9)	81 (43.1)	0.057
Blood culture requested	696 (51.0)	668 (49.0)	0.448
Primary bloodstream infections	77 (11.1)	60 (9.0)	0.225
Total patient days	9583 (47.1)	10 751 (52.9)	< 0.001
Central line-associated bloodstream infections	55 (56.7)	42 (43.3)	0.187
Total device days	3970 (46.2)	4625 (53.8)	< 0.001
Central line-associated bloodstream infections per 1000 device days (95% CI)	13.85 (10.44–18.03)	9.08 (5.81–11.27)	0.0378

CI: confidence interval; WHO: World Health Organization.

^a^ Values are no. (%) if not otherwise stated.

Note: the pre-intervention period ran from December 2022 to April 2023. The post-intervention period ran from May 2023 to October 2023.

**Fig. 1:**
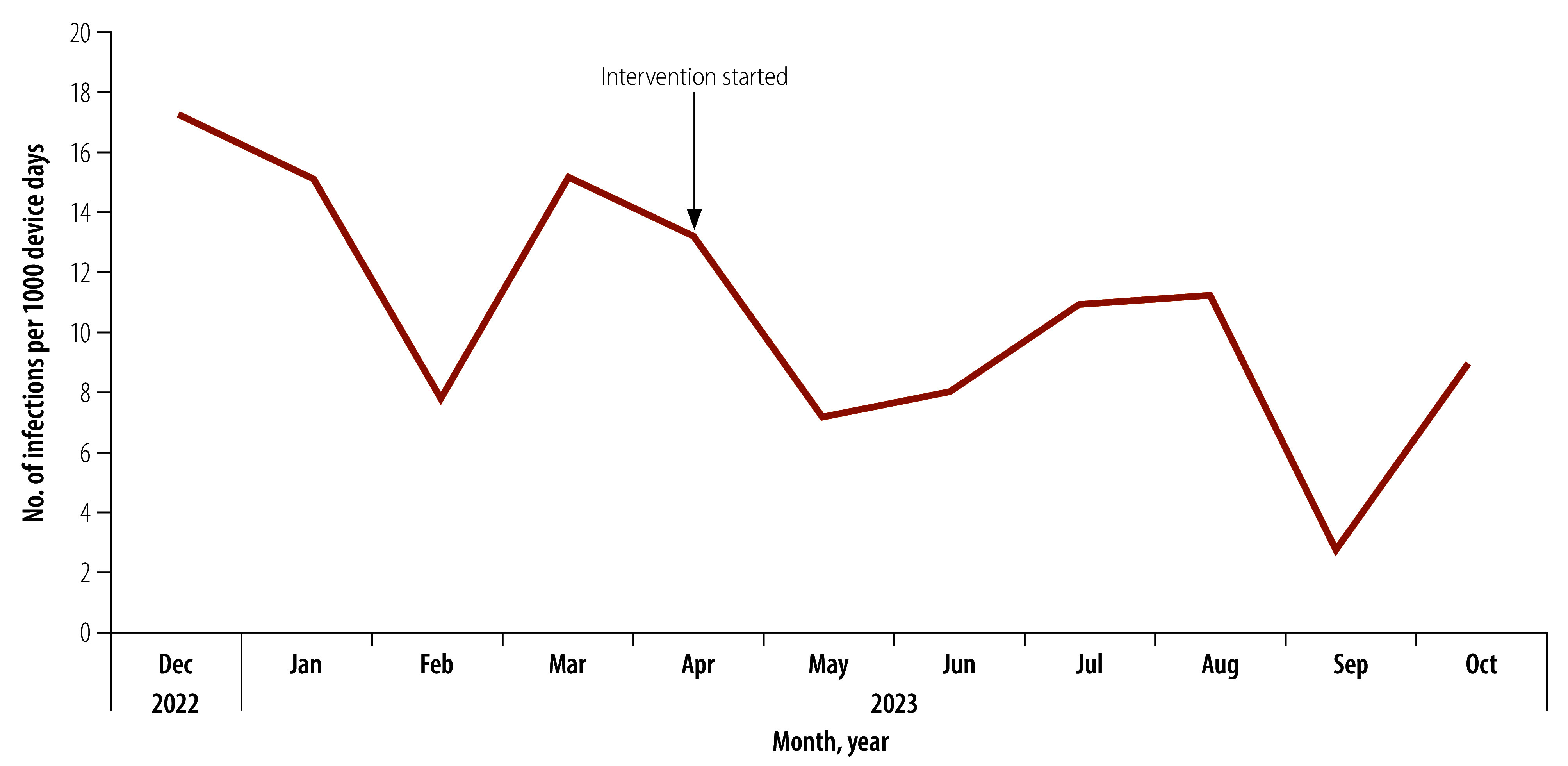
Monthly trend of central line-associated bloodstream infection in a neonatal intensive care unit, Egypt, December 2022 – October 2023

The central line-associated bloodstream infection rate decreased from 13.85 infections per 1000 device days (95% confidence interval, CI: 10.44–18.03) before the intervention to 9.08 infections per 1000 device days (95% CI: 5.81–11.27). Fig. 1 presents the trends of central line-associated bloodstream infections over the study period.

Nurses’ hand hygiene compliance increased from 58% (70/120) at the beginning of the study to 71% (88/124) at the end of the study period, whereas for the doctors, compliance increased from 64% (58/91) to 67% (67/100). 

## Lessons learnt

Implementing infection prevention and control measures to reduce the incidence of central line-associated bloodstream infections is crucial for reducing neonatal morbidity and mortality rates and decreasing hospital length of stay and associated costs.^8^ The multimodal strategy implemented strengthened infection prevention and control practices in our neonatal intensive care unit. This increased compliance in turn led to a decrease in central line-associated bloodstream infections. All these changes became embedded in behaviours and practices at the unit, and a part of quality improvement actions that ensure sustainability and safety culture at the neonatal intensive care unit (Box 1).

Box 1 **Summary of main lessons learnt**

- Mobilizing resources is essential for effective infection prevention and control, and reducing health care-associated infections.
- Leadership engagement helps promote hand hygiene and allocate the resources for infection prevention and control measures.
- WHO's multimodal strategy helps in changing the attitude and practices related to infection prevention control, ultimately improving patient outcomes and promoting a safe health-care environment.

Several factors contributed to decreased central line-associated bloodstream infections. First, the creation of a multidisciplinary team and regular meetings with unit decision-makers made staff members more involved in the infection prevention and control challenges. These decisions had a positive impact on the infection prevention and control practices overall and led to a decrease in infections. Without a dedicated budget for infection control activities and centralized resource and product distribution, the store was often out of the supplies we needed. Thus, leadership support was a cornerstone in the successful implementation, by ensuring product availability, engaging staff members and raising awareness about the importance of best infection prevention and control practices, especially hand hygiene and surface disinfection, in safeguarding against infections.

Changing doctors’ perceptions and attitudes towards the role of infection prevention and control in improving patient outcomes was challenging. However, implementing a multimodal strategy and creating a multidisciplinary team helped engage doctors as active key players, particularly by involving them in decision-making and influencing practices among junior doctors and residents.

These findings show that multidisciplinary and multimodal approaches reduce central line-associated bloodstream infections by educating health workers and monitoring their adherence to hygiene practices. These approaches also ensure good communication and collaboration between health workers. Implementing these approaches in other hospitals may improve patient safety and reduce health care-associated infections.
